# Therapeutic Potential of αS Evolvability for Neuropathic Gaucher Disease

**DOI:** 10.3390/biom11020289

**Published:** 2021-02-15

**Authors:** Jianshe Wei, Yoshiki Takamatsu, Ryoko Wada, Masayo Fujita, Gilbert Ho, Eliezer Masliah, Makoto Hashimoto

**Affiliations:** 1 Tokyo Metropolitan Institute of Medical Science, 2-1-6 Kamikitazawa, Setagaya-ku, Tokyo 156-0057, Japan; jswei@henu.edu.cn (J.W.); takamatsu-ys@igakuken.or.jp (Y.T.); wada-rk@igakuken.or.jp (R.W.); fujita-ms@igakuken.or.jp (M.F.); 2 Institute for Brain Sciences Research, School of Life Sciences, Henan University, Kaifeng 475004, China; 3 PCND Neuroscience Research Institute, Poway, CA 92064, USA; giho@pcndneurology.com; 4 Division of Neurosciences, National Institute on Aging, National Institutes of Health, Bethesda, MD 20892, USA; eliezer.masliah@nih.gov

**Keywords:** Gaucher disease (GD), Parkinson’s disease (PD), autosomal recessive, α-synuclein (αS), evolvability, antagonistic pleiotropy, β-synuclein (βS)

## Abstract

Gaucher disease (GD), the most common lysosomal storage disorder (LSD), is caused by autosomal recessive mutations of the glucocerebrosidase gene, *GBA1*. In the majority of cases, GD has a non-neuropathic chronic form with adult onset (GD1), while other cases are more acute and severer neuropathic forms with early onset (GD2/3). Currently, no radical therapies are established for GD2/3. Notably, GD1, but not GD2/3, is associated with increased risk of Parkinson’s disease (PD), the elucidation of which might provide a clue for novel therapeutic strategies. In this context, the objective of the present study is to discuss that the evolvability of α-synuclein (αS) might be differentially involved in GD subtypes. Hypothetically, aging-associated PD features with accumulation of αS, and the autophagy-lysosomal dysfunction might be an antagonistic pleiotropy phenomenon derived from αS evolvability in the development in GD1, without which neuropathies like GD2/3 might be manifested due to the autophagy-lysosomal dysfunction. Supposing that the increased severity of GD2/3 might be attributed to the decreased activity of αS evolvability, suppressing the expression of β-synuclein (βS), a potential buffer against αS evolvability, might be therapeutically efficient. Of interest, a similar view might be applicable to Niemann-Pick type C (NPC), another LSD, given that the adult type of NPC, which is comorbid with Alzheimer’s disease, exhibits milder medical symptoms compared with those of infantile NPC. Thus, it is predicted that the evolvability of amyloid β and tau, might be beneficial for the adult type of NPC. Collectively, a better understanding of amyloidogenic evolvability in the pathogenesis of LSD may inform rational therapy development.

## 1. Introduction

**Figure 1:**
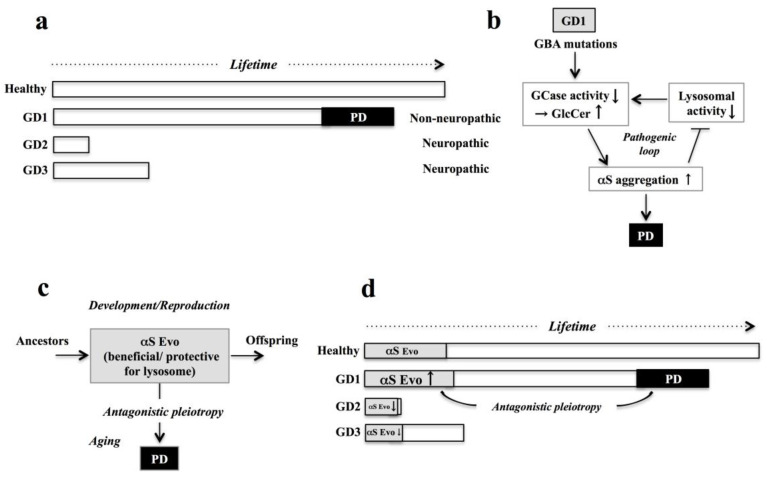
Involvement of α-synuclein (αS) evolvability in the association of Parkinson’s disease (PD) with Gaucher disease (GD). (**a**) Schematics of three subtypes of GD; GD1–3. (**b**) A “bidirectional feedback loop” hypothesis, the most widely accepted rationale explaining the pathophysiological mechanism underlying the GD–PD association [4]. Conventionally, it was thought that accumulation of glucocerebroside (GluCer) caused by the compromised glucocerebrosidase (GCase) activity due to mutations of glucocerebrosidase (GBA) might result in stimulation of aggregation of αS, which then exacerbates lysosomal function, thus leading to formation of a vicious cycle of neurodegeneration until manifestation of PD. (**c**) Our amyloidogenic evolvability hypothesis; in the development/reproduction stage, αS evolvability (Evo) is lysotrophic and lysoprotective against the multiple stressors caused by autophagy-lysosomal dysfunction, and the stress information might be transgenerationally delivered to offspring. On the other hand, neurodegenerative diseases such as PD that are associated with autophagy-lysosomal dysfunction might be manifested as an antagonistic pleiotropy mechanism in aging. (**d**) Schematics of the differential role of αS evolvability in subtypes of GD. In GD1, αS Evo is upregulated to mitigate the multiple stressors caused by autophagy-lysosomal dysfunction in development/reproduction, but PD is later manifested through the antagonistic pleiotropy mechanism. By virtue of increased αS evolvability, GD1 is non-neuropathic. In contrast, αS Evo is suppressed in GD2/3, with GD2 being stronger than GD3. Consequently, GD2/3 are neuropathic and life expectancy is short, with GD2 being severer than GD3.

Gaucher disease (GD) is the most common lysosomal storage disorder (LSD) [1]. Due to autosomal recessive mutations of glucocerebrosidase gene 1 (*GBA1*) encoding the lysosomal hydrolase that is responsible for the degradation of glucosylceramide (GlcCer), GlcCer accumulates intracellularly, leading to a form of sphingolipidosis [1]. Consequently, patients typically manifest hepatosplenomegaly, hematological changes, anemia, and orthopedic complications [2]. Depending on the presence or absence of neurological involvement and on its overall severity, three different subtypes of GD may be recognized (Figure 1a) [3,4]. GD1 is the most common (~90%) non-neuropathic form with adult onset. The median age at diagnosis is 28 years of age, and life expectancy is mildly decreased [4]. In contrast, GD2 and GD3 are acute and sub-acute, respectively. GD2 displays severe neurological involvement, leading to death within the first years of life in small children, while GD3 is a chronic neuronopathic form that exhibits systemic involvement of varying degree with at least one neurological manifestation. This group develops the disease somewhat later, but most patients die before their 30th birthday [4]. To date, the molecular mechanisms underlying the different subtypes of GD are unclear.

Notably, GD1 may increase the risk of sporadic Parkinson’s disease (PD) characterized by enhanced α-synuclein (**α**S) pathology in aging (Figure 1a), although the mechanisms are elusive [4,5]. Given that various harmful molecules may be released through lysosomal membrane permeabilization (LMP) [6,7], αS evolvability might be important particularly under stressful conditions such as LSD. In this context, the main objective of the present study was to discuss the possibility that αS evolvability might be differentially involved in each GD subtype. Predictably, increased evolvability might protect lysosomes in the brain with GD1, which might later dysregulate the autophagy-lysosomal pathway through the antagonistic pleiotropy mechanism, leading to PD. In contrast to GD1, the decrease in αS evolvability in both GD2/3 might account for the severity of these diseases. If such a prediction might be the case, increasing αS evolvability through a decrease in the buffering effect of β-synuclein (βS) might be therapeutically effective for GD2/3. Collectively, a better understanding of αS evolvability in GD pathogenesis may inform rational therapy development.

## 2. Conventional View of the Relationship between GD and αS Pathology

Although the etiology of sporadic PD is obscure, recent research has revealed that heterozygous mutations of *GBA1*, encoding for lysosomal enzyme GCase, might increase the risk of PD in considerable cases [8,9,10].

### 2.1. Association of PD with GBA1 Mutations

Since the comorbidity of Parkinsonism among GD patients and *GBA1* mutation carriers was first recognized in the clinics [11], the association of the *GBA1* mutation with PD development has been independently reported by many association studies [12]. In particular, a meta-analysis of data collected from 16 centers established that there was a strong association between *GBA1* mutations and PD [13]. Thus, it was concluded that *GBA1* mutations were a major genetic risk factor for sporadic PD.

### 2.2. Proposed Mechanism of Association between GD and PD

Identification of the pathological mechanisms underlying *GBA1*-associated Parkinsonism might improve our understanding of the pathophysiology and treatment of GD in aging (Figure 1a). Interestingly, however, the mechanisms underlying that process remain unclear/elusive, even though a number of hypotheses have already been published [4]. It was predicted that αS aggregation might be promoted due to a “gain-of-function” of *GBA1* mutations [14]. It was also described that substrate accumulation due to enzymatic “loss-of-function” caused by *GBA1* mutations might affect processing and clearance of αS [15]. The most popular view is the “bidirectional feedback loop” hypothesis [16], proposing that accumulation of GlcCer by the compromised activity of GCase due to the mutations of *GBA1* might stimulate the aggregation of αS (Figure 1b). Then, the increased neurotoxic αS might exacerbate lysosomal functions, including GCase activity, leading to the formation of a vicious cycle of neurodegeneration in aging (Figure 1b) [17,18].

Accordingly, PD is supposed to be situated downstream from GD. Since heterozygotes of GD are asymptomatic, GD and its downstream PD might be not selected out in evolution. Furthermore, GD 2/3 might not be associated with PD because life lengths of these neuropathic types of GD are too short for manifestation of PD. Although such a view is plausible, it cannot explain why so many mutations are accumulated in the *GBA1*.

## 3. Comorbidity of GD1 with PD from Viewpoint of αS Evolvability

The majority of αS studies have so far focused on the neurotoxic aspects of αS relevant to neurodegenerative diseases. However, there are indeed several studies suggesting that αS might also be beneficial, including its evolvability, a potential physiological function of amyloidogenic proteins (APs).

### 3.1. Physiological Functions of αS

αS was previously identified as synelfin, the avian form of αS, which might be essential for bird song memory formation during a critical period in development [19]. Thus, αS might play a crucial role in learning and memory during mammalian neurodevelopment. Consistent with this notion, αS was shown to cooperate with cysteine string protein α, the co-chaperone that is essential for neuronal survival, synaptic protection, and preventing neurodegeneration [20]. Collectively, these results in vivo suggest that αS might play a beneficial role in development. In addition, it was previously shown that αS might be involved in oxidative stress-resistance. αS was shown to protect against oxidative stress in vitro [21,22]. Indeed, it was recently shown that αS prevented the formation of oxidative stress-induced formation of spherically-shaped and hyperpolarized mitochondria, termed “mitospheres”, leading to suppression of apoptosis under oxidative stress conditions [23]. Thus, αS may be physiologically beneficial in the brain.

### 3.2. Evolvability of αS

So far, the physiological function of APs relevant to neurodegenerative diseases, such as amyloid β (Aβ) and αS, has been obscure. In this regard, yeast prion is worth noting, in which alteration of the aggregation states of APs act as a genetic switch in response to the diverse environmental conditions [24]. Given the analogy that both the yeast and the aged brain are in stressful conditions, we recently proposed that the evolvability of APs might play an important role in the human brain [25]. Evolvability is defined as the capacity of an organism for adaptive evolution [26]. More specifically, evolvability consists of two steps; to generate a genetic diversity against environmental conditions including stressors, and to deliver the information to progeny [26]. As described above, αS might be involved in stress-resistance. Because of their intrinsically disordered structures [27], APs including αS may show a diverse morphology in response to multiple stressors, such as oxidative stress, kindling, physical stress, and neurotoxicity, followed by formation of the stress-specific AP protofibrils, which might confer resistance against stressors in the parental brain. The AP protofibrils are then subjected to transgenerational transmission via germ cells in a prion-like fashion [28]. By virtue of the stress information of protofibrils derived from parental brains, an offspring’s brain can better cope with forthcoming stresses that otherwise would lead to the onset of neurodevelopmental disorders [28]. Yet, as a negative consequence, neurodegeneration may manifest in the parental brain through the antagonistic pleiotropy mechanism in aging [28]. Thus, it is predicted that amyloidogenic evolvability may be an evolutionally beneficial physiological function.

### 3.3. Possible Relevance of αS Evolvability to Pathogenesis of GD

As described earlier, LMP underlies lysosomal release of various harmful molecules, including reactive oxygen species, proteases, and lysosomal membrane lipid compositions. Under such stressful conditions, stress information by αS transmission, conferring stress resistance, might be important to deliver to offspring. Thus, αS evolvability could be regarded as the inheritance of acquired characteristics related to environmental stresses (Figure 1c) [25]. According to such a view, PD manifested in the aging stage of GD1 might be interpreted as an antagonistic pleiotropy phenomenon of the increased αS evolvability, which is lysosome-protective (Figure 1c,d). Considering that the onset of GD1 is approximately in the late 20s, αS evolvability can be transmitted to offspring and is therefore evolutionally beneficial. However, PD may manifest as a stable phenotype during the aging of patients with GD1 (Figure 1c). Furthermore, given that GD1 is sometimes associated with other α-synucleinopathies, such as dementia with Lewy bodies (DLB) and multiple system atrophy [29], similar mechanisms might be applied to other members of the α-synucleinopathies. In contrast, neuropathies become severe and the length of life is extremely short in GD 2/3 in which αS evolvability might be decreased (Figure 1c). From the viewpoint of amyloid evolvability, it is assumed that a number of GBA mutations might have accumulated as a result of adaptation.

### 3.4. βS as Buffer against αS Evolvability

The precise mechanism by which the alteration of αS evolvability might be differential depending on the type of GD is unclear. Among various factors that might affect αS evolvability in the pathogenesis of GD, βS, a member of the αS family of peptides, might be potentially interesting. βS is a non-amyloidogenic homologue of αS due to the natural deletion of the central hydrophobic domain, known as NAC: non-Aβ component of Alzheimer’s disease (AD) amyloid [30]. Given that αS aggregation is inhibited by wild type βS [31,32], βS might exert a buffering effect on αS evolvability. Given that the risk of PD occurs in only a subset of GD1 cases, it is possible that there might be other contributory factors. In this regard, one may speculate that expression of βS might be decreased in GD1, while it is increased in GD 2/3.

### 3.5. Experimental Support of Pleiotropic Effects of αS in Terms of Lysosomal Activity

**Figure 2:**
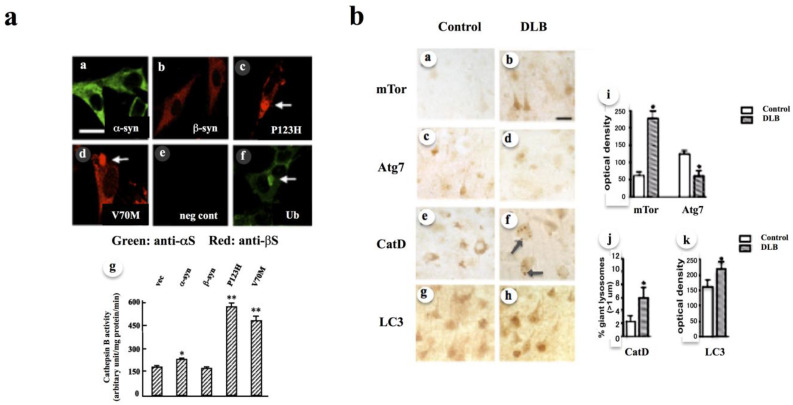
Dual effects of αS on lysosome activity in cells and human brain. (**a**) Up-regulation of lysosomal activity in cells overexpressing mutant αS [33]. Overexpression of A53TαS in B103 neuroblastoma cells resulted in increased lysosomal activity (a, g). Notably, the increase in lysosome activity appeared more prominently in mutants (P123H and V70M) βS, but not in wild type βS (b–d, g). Immunofluorescence with confocal microscopy (green: anti-αS; red: anti-βS) was performed in a-e, while cathepsin B activity was measured using fluorogenic cathepsin B substrate. Fluorogenic intensity of each time point was plotted, and the slope was calculated. Data are shown as means ± SD (*n* = 4). * *p* < 0.05, ** *p* < 0.01 versus vector-transfected cells. (**b**) Selective molecular alterations in the autophagy pathway in patients with dementia with Lewy bodies (DLB) [34]. Vibratome sections from the temporal cortex of non-demented controls and DLB patients were analyzed by immunohistochemistry. Representative sections from control and DLB brains were immunolabeled with antibodies against mTor (a, b), Atg7 (c, d), CatD (e, f), and LC3 (g, h). Semi-quantitative image analysis reveals a significant increase in mTor levels and a reduction in Atg7 levels in DLB patients compared to controls (i). Similarly, both CatD (j) and LC3 (k) immunoreactivity levels in DLB brains were significantly increased compared to those of controls. Pyramidal neurons in DLB cases show enlarged CatD-immunoreactive lysosomes (arrows). Scale bar in panel (b) represents 20 µm in all microscopy images. * *p* < 0.05 compared to non-demented controls in one-way ANOVA with post-hoc Dunnett’s test.

Previous experimental results are consistent with the role of αS evolvability in GD1. Briefly, B103 neuroblastoma cells expressing αS exhibited increased lysosomal activity (Figure 2a) [33], indicating that αS could be beneficial for lysosomes. Conversely, αS might be detrimental to the lysosomal-autophagy pathway in DLB during aging based on the expression levels of various molecules, including mTor, Atg7, cathepsin D (CatD), and LC3 (Figure 2b) [34], suggesting that αS could be detrimental to lysosomes in aging. Collectively, it is predicted that the antagonistic pleiotropy relationship between αS evolvability and α-Synucleinopathies might be at least in some parts through the dual effects of αS on lysosomes. Of considerable interest, the increase in lysosomal activity was drastic in cells expressing DLB-linked βS mutations (P123H, V70M) (Figure 2a) [33,35]. Supposing that the βS mutations might increase αS evolvability, it is reasonable that these mutations might manifest as DLB through the antagonistic pleiotropy mechanism in aging [36]. Thus, βS might be involved in both stimulation and suppression of amyloidogenic evolvability and neurodegeneration.

## 4. Application of Evolvability Hypothesis to Other LSDs

Since LSD comprises about 50 rare inherited metabolic disorders that are caused by lysosomal dysfunction as a consequence of deficiency of a single enzyme [1], it is natural to predict that amyloid evolvability might also be involved in other LSD.

### 4.1. Niemann-Pick Type C (NPC)

NPC is a rare progressive genetic disorder characterized by an inability of the body to transport cholesterol and other lipids inside of cells because of the autosomal recessive gene mutation of either *NPC1* or *NPC2*. Consequently, the abnormal accumulation of these lipids in various tissues of the body, including brain, damages the affected areas [37,38]. The age of onset of NPC is highly variable, ranging from a fatal disorder within the first few months after birth (early infantile type) to a late-onset, chronic, progressive disorder that remains undiagnosed well into adulthood (adult type) [37,38]. Most cases are detected during childhood and progress to cause life-threatening complications by the second or third decade of life [37].

### 4.2. NPC and Amyloidogenic Evolvability

Interestingly, NPC has been well investigated in terms of the comorbidity with AD. NPC is histologically associated with AD pathologies, including neurofibrillary tangle formation and Aβ deposition in adulthood [39,40], suggesting that evolvability of APs, including tau and Aβ, might play a role.

Given the analogy with GD, it is thought that accumulation of cholesterol by the compromised activity of *NPC1* (or *NPC2*) due to gene mutations might stimulate the aggregation of Aβ/tau. Then, the increased neurotoxic Aβ/tau might exacerbate lysosomal functions, including NPC1 (or NPC2) activity, leading to the formation of a vicious cycle of neurodegeneration in aging. According to our view of the “evolvability hypothesis,” the increased APs (Aβ/tau) evolvability might mitigate the disease severity in development, while leading to the manifestation of AD through antagonistic pleiotropy in the adult type. Conversely, the decreased APs evolvability in development might be relevant to the increased disease severity in the early infantile type.

Notably, the results of animal experiments are consistent with the current hypothesis. For instance, cross-breeding of an amyloid precursor protein knockout mouse with a mouse model of NPC disease exhibited exacerbation of its phenotypes, suggesting that absence of Aβ evolvability failed to rescue the phenotype of NPC1 mouse [41]. Similarly, NPC1/tau double-null mice exhibited an exacerbated NPC phenotype, including severe systemic manifestations, and died significantly earlier than NPC1 single-null mutant mice [42].

Besides NPC, various types of LSDs, such as mucopolysaccharidoses (MPS), sialidosis, and Krabbe disease, have been characterized by amyloidosis and α-synucleinopathies [43]. Thus, it is predicted that amyloidogenic evolvability may underlie the association of neurodegenerative diseases with LSDs.

## 5. βS as Therapeutic Target

So far, symptomatic treatments, such as enzyme replacement therapy (ERT) and substrate reduction therapy (SRT), have been developed successfully for GD1 [44], while no radical treatments are available for GD2/3. Thus, it is expected that the concept of αS evolvability might provide a clue for a novel therapy, especially for GD2/3.

### 5.1. Conventional Therapy

ERT is mainly provided for GD1 using recombinant GCase, which is not used for GD2/3 because this protein does not pass through the blood-brain barrier [45]. Similar strategies are applied to other non-neuropathic types of LSD to replace the deficient enzyme with artificial ones [45]. These medications are given intravenously to outpatients, and may occasionally cause an allergic or hypersensitivity reaction to treatment [44]. SRT is an alternative oral treatment for GD1 to reduce the rate of biosynthesis of glycosphingolipids to offset the catabolic defect [44]. Less frequently, other treatments such as bone marrow transplant are performed for GD1 to remove and replace blood-forming cells that have been damaged [44], but this is of little benefit to GD2/3. Collectively, there are currently no effective treatments for GD2/3.

### 5.2. Evolvability-Based Novel Therapy

**Figure 3:**
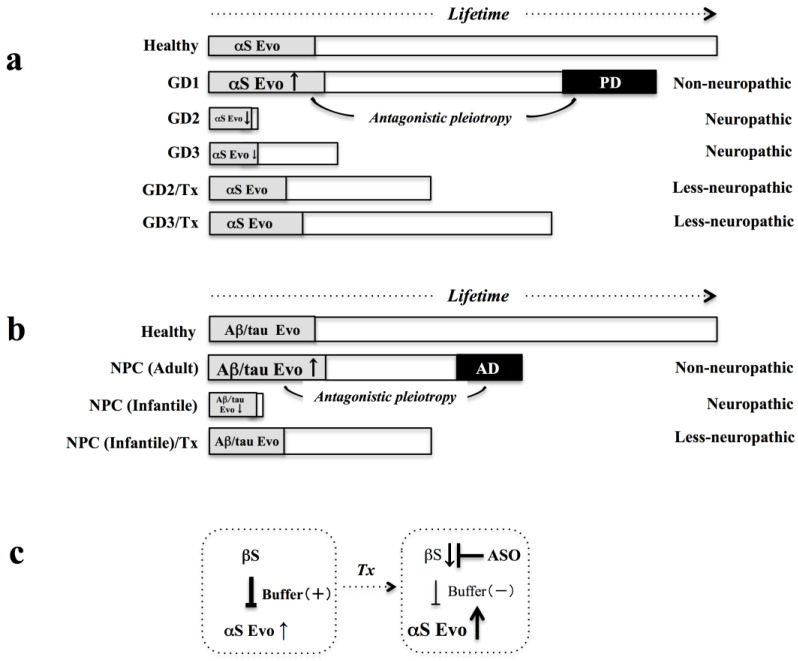
Therapy strategy of LSDs based on αS evolvability. (**a**) Diagram of the increase of αS Evo in GD2/3. If decreased Evo αS is causative for GD2/3, it is predicted that increasing αS Evo might be beneficial. Accordingly, the severity of GD2/3 neuropathy might be improved with extended life expectancy. (**b**) Diagram of the differential role of Aβ/tau evolvability in subtypes of NPC. In the adult type of NPC, Aβ/tau Evo is upregulated to mitigate the multiple stressors caused by autophagy-lysosomal dysfunction, but AD is later manifested through the antagonistic pleiotropy mechanism. The life expectancy of NPC (adult type) patients is shorter than that of healthy controls. In contrast, Aβ/tau Evo is suppressed in NPC (early infantile type), and life expectancy is very short. By therapeutically increasing Aβ/tau Evo, the severity of NPC (early infantile type) might be improved with extended life expectancy. (**c**) Therapy strategy based on αS evolvability. Since it is predicted that βS has a buffering effect on αS evolvability, αS evolvability might be increased by suppressing βS expression by ASO (Therapy: Tx).

If the severe phenotypes in GD2/3 compared to the mild symptoms in GD1 might be attributed to the decreased αS evolvability, it is reasonable to predict that increasing αS evolvability might be therapeutic for GD2/3 (Figure 3a). For this purpose, one possible strategy would be to supply exogeneous αS, especially the aggregate-prone species. However, considering that active immunotherapy of amyloid β for AD patients caused encephalomyelitis [46], the injection of αS recombinant proteins might be also harmful. Alternatively, αS evolvability might be increased by reducing βS expression. This could be performed by βS antisense oligonucleotide (ASO) at the mRNA level (Figure 3c) [47]. In this regard, ASO has been well established and has been successfully used for various diseases, including spinal muscular atrophy [48]. A similar strategy could be applicable to NPC (Figure 3b) and perhaps other LSDs, including mucopolysaccharidoses (MPS) and Krabbe disease. In case of NPC, evolvability of APs, such as Aβ and tau, might be upregulated by downregulating the βS expression (Figure 3c). In support of this possibility, βS was shown to associate with αS and Aβ in vitro [49]. It is unclear whether βS binds with tau. However, given that αS stimulates tau aggregation in vivo [50], βS might either directly or indirectly suppress tau aggregation/evolvability. Obviously, the current hypothesis will require experimental demonstration. For this purpose, mice and small fish models [51,52,53,54] might be suitable considering the endogenous expression of βS.

However, it is possible that therapeutically increased amyloidogenic evolvability in young age might lead to neurodegenerative diseases through the antagonistic pleiotropy mechanism in aging. Furthermore, since amyloidogenic evolvability also be involved in various cancer phenotypes, such as cell proliferation, resistance against medical treatments and metastasis [55,56], there is concern that the therapeutic increase of amyloidogenic evolvability in GD might stimulate cancer. Thus, these possibilities should be well recognized, and patients must be carefully followed-up after anti-βS treatment.

## 6. Conclusions

Based on previous studies describing that GD1 is a major risk factor of sporadic PD, it is generally thought that GD1 might be situated upstream of the pathogenesis of PD. However, distinct from such a conventional view, we propose the “evolvability hypothesis,” in which a physiological role of αS evolvability is supposed, which is protective of lysosomes, without which neuropathy might be promoted. Instead, the risk of PD might be increased through an antagonistic phenomenon in aging. Thus, it is predicted that the comorbidity of GD1 with PD might be attributed to the increased level of αS evolvability in development/reproduction.

Our view is attractive from a therapeutic viewpoint. Compared to GD1, there are currently fewer radical therapies established for GD2/3. If the decrease of αS evolvability might be causative of GD2/3, increasing αS evolvability might be therapeutic in these devastating diseases. Provided that βS may act as a buffer against αS evolvability, decreasing βS expression by ASO might be efficient to increase αS evolvability. Finally, it is interesting to speculate that an essentially similar view of αS evolvability could be applicable not only to GD but also to other LSDs, such as NPC, MPS, sialidosis, and Krabbe disease. Thus, a unified understanding of the mechanism and therapy of LSDs might be expected from a viewpoint of amyloidogenic evolvability.
